# Why learning progress needs absolute values: Comment on Poli et al. (2024)

**DOI:** 10.1111/ejn.16635

**Published:** 2024-12-05

**Authors:** Augustin Chartouny, Benoît Girard, Mehdi Khamassi

**Affiliations:** ^1^ Institut des Systèmes Intelligents et de Robotique Sorbonne Université–CNRS Paris France

**Keywords:** curiosity, exploration, surprise

In a recent issue of TiCS, Poli et al. (2024) reviewed the latest developments of computational models of curiosity in cognitive neuroscience and promoted learning progress as a key computational mechanism for optimal environmental exploration. Here, we want to emphasize results from the machine learning literature showing that their mathematical formula of learning progress may be sub‐optimal. We present an alternative formulation of learning progress with absolute values, solving this problem. Learning progress with absolute values provides further insights into the decision‐making mechanisms that may underlie exploration. It also demonstrates the need for new experiments to disambiguate all the existing interpretations of learning progress.

Learning progress promotes exploration depending on how much an agent (e.g., human, animal or robot) is learning (Oudeyer et al., 2007). Agents should explore options for which they progress quickly because there is potentially more to learn. In contrast, agents should ignore options for which they have not made progress, as there might be nothing new to learn. Poli et al. suggest that a good proxy for learning progress is the change in prediction errors over time (Oudeyer et al., 2007). With this formulation, a decrease in prediction errors indicates that the agent is currently learning and should keep exploring to continue improving. Conversely, an increase in prediction errors makes the learning progress negative and should result in the agent avoiding options that become unpredictable. However, it has been shown in the machine learning literature that exploration should increase when prediction errors increase, either after a task change to adapt to the new task (Chartouny et al., 2024) or when the agent starts forgetting how to solve the task (Colas et al., 2019). Authors commonly use a formulation of learning progress with absolute values to induce exploration equally between increases and decreases of performance (Chartouny et al., 2024; Colas et al., 2019).

Learning progress with absolute values seems more efficient from a machine learning perspective, but we argue that it also seems more promising in explaining human exploration. With absolute values, increases in prediction error induce curious behaviours. This is consistent with experimental results showing that humans explore more when tasks become suddenly surprising. For example, Collins and Koechlin (2012) reported that humans' exploratory response rates went from 5% in a stable environment to 40% three or four trials after a task change and slowly decreased back to 5% as the surprise vanished. Furthermore, Stahl and Feigenson (2015) demonstrated that infants explore and learn more about the properties of objects that surprise them. Finally, learning progress with absolute values explained significantly better human behaviour and pupil size variation than learning progress without absolute values in an arithmetic task with summations of varying difficulty (Sayalı et al., 2023). Thus, models of learning progress with absolute values have proven to be useful in cognitive neuroscience. Further research is required to see whether they more generally account for human exploratory behaviour in various situations and whether neural correlates of such a mechanism can be found in brain activity.

As highlighted by Poli et al. (2024), learning progress has limitations. However, their claim that learning progress does not provide ‘how useful a given activity is to the agent's goal’ may be misleading. The article they cite praises the role of learning progress in goal‐directed exploration (Molinaro & Collins, 2023). Molinaro and Collins state that ‘studies of human behavior have confirmed the prominent role of learning progress in dictating which goals people end up pursuing’. Thus, learning progress permits generating goals of increasing difficulty and achieving them. Moreover, one of the main applications of learning progress in the reinforcement learning literature is to learn tasks with multiple goals (Colas et al., 2022). This illustrates that learning progress is central to understanding goal‐oriented behaviours. However, a formulation of learning progress as differences of prediction errors is not appropriate to study goal‐oriented behaviours. Thus, authors use alternative formulations such as measures of competence (Colas et al., 2019). This demonstrates that there is no canonical learning progress formula, that there is a need for a taxonomy of all the learning progress formulations in the literature and that more experiments with humans should compare different learning progress formulations.

The diversity of interpretations of learning progress comes from the fact that learning progress is a simple heuristic: If curiosity is a desire for knowledge (Kang et al., 2009), curious humans should focus on options that maximize what they learn. Based on this observation, many formulations of learning progress have been introduced, such as the decrease in prediction errors (Oudeyer et al., 2007; Poli et al., 2024), the absolute difference in percentage correct (Sayalı et al., 2023), the absolute difference in competence (Colas et al., 2019) and the variation of precision of an internal model (Chartouny et al., 2024). All these formulations share the idea that learning progress computes a variation of performance metrics, which permits to avoid tasks that are either trivial or impossible. Future studies should investigate how well each formulation explains human exploration on the same task.

## AUTHOR CONTRIBUTIONS

**Augustin Chartouny:** Writing—original draft. **Benoît Girard:** Supervision; validation; writing—original draft. **Mehdi Khamassi:** Supervision; validation; writing—original draft.

## CONFLICT OF INTEREST

The authors declare no conflict of interest.

### PEER REVIEW

The peer review history for this article is available at https://www.webofscience.com/api/gateway/wos/peer-review/10.1111/ejn.16635.
